# Supplementary material for: Biomechanics Analysis of the Firefighters’ Thorax Movement on Personal Protective Equipment during Lifting Task Using Inertial Measurement Unit Motion Capture
Source: Int J Environ Res Public Health. 2022 Oct 31;19(21):14232. doi: 10.3390/ijerph192114232 (PMC9658051; doi:10.3390/ijerph192114232)
Supplement: Supplementary file 1 [file ijerph-19-14232-s001.zip › ijerph-1891230-supplementary (1).pdf]

File S1: SELF ASSESSMENT MUSCULOSKELETAL PAIN / DISCOMFORT SURVEY FORM (Refer to Part 2.1)

Instruction:

1. Tick (√) on any body parts (Column A) if you feel discomfort/pain during your work in the last 12 months
2. For those body parts you were feeling pain/discomfort, tick (√) (Column B) if in your opinion, the pain is due to your work.

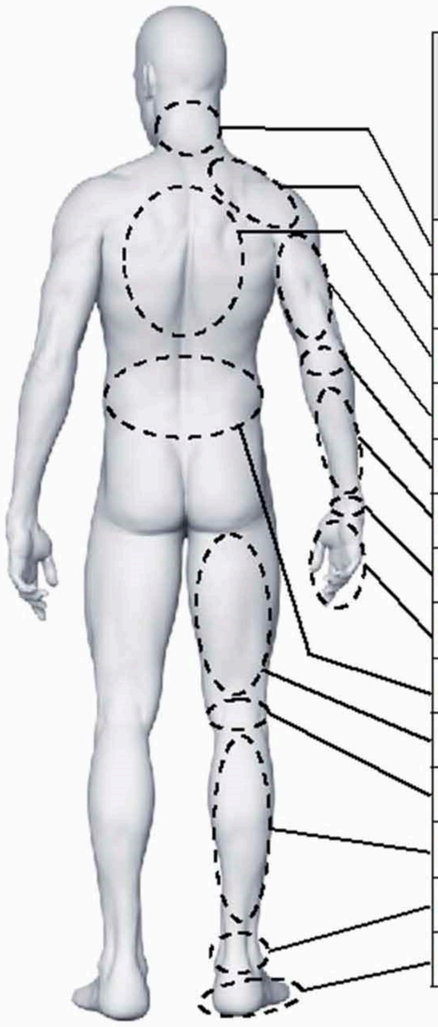

Body Parts	A<br>I have pain/ discomfort in the following body parts.		B<br>I think the pain/ discomfort comes from work.	
Neck				
Shoulder				
Upper back				
Upper arm	L	R	L	R
Elbow	L	R	L	R
Lower arm	L	R	L	R
Wrist	L	R	L	R
Hand	L	R	L	R
Lower back				
Thigh	L	R	L	R
Knee	L	R	L	R
Calf	L	R	L	R
Ankle	L	R	L	R
Feet	L	R	L	R

Name: ____________________ Staff ID No.: ____________________

Department: ____________________ Job tasks ____________________

Contact No.: ____________________ Email: ____________________

Date: ____________________

(Do not write anything in the below section. To be filled by trained person only)

Is/Are the symptom(s) work related? Yes ☐ No ☐

Comments: ______________________________________

____

Name: ____________________ Signature and stamp:

Date: ____________________
